# Biomass Pyrolysis-Derived Biochar: A Versatile Precursor for Graphene Synthesis

**DOI:** 10.3390/ma16247658

**Published:** 2023-12-15

**Authors:** Karla Plenča, Sara Cvetnić, Helena Prskalo, Marin Kovačić, Matija Cvetnić, Hrvoje Kušić, Zvonimir Matusinović, Marijana Kraljić Roković, Boštjan Genorio, Urška Lavrenčič Štangar, Ana Lončarić Božić

**Affiliations:** 1 Faculty of Chemical Engineering and Technology, University of Zagreb, Marulićev trg 19, 10000 Zagreb, Croatia; kplenca@fkit.hr (K.P.); mkovacic@fkit.hr (M.K.); mcvetnic@fkit.hr (M.C.); mkralj@fkit.hr (M.K.R.); 2 Department for Safety and Protection Engineering, Karlovac University of Applied Sciences, Trg J.J. Strossmayera 9, 47000 Karlovac, Croatia; sara.cvetkovic789@gmail.com (S.C.); prskalohelena5@gmail.com (H.P.); zvonimir.matusinovic@vuka.hr (Z.M.); 3 Department for Packaging, Recycling and Environmental Protection, University North, Trg dr. Žarka Dolinara 1, 48000 Koprivnica, Croatia; 4 Faculty of Chemistry and Chemical Technology, University of Ljubljana, Večna pot 113, SI-1000 Ljubljana, Slovenia; bostjan.genorio@fkkt.uni-lj.si (B.G.); urska.lavrencic.stangar@fkkt.uni-lj.si (U.L.Š.)

**Keywords:** pyrolysis, biomass, biochar valorization, graphene synthesis

## Abstract

Graphene, a two-dimensional carbon allotrope with a honeycomb structure, has emerged as a material of immense interest in diverse scientific and technical domains. It is mainly produced from graphite by mechanical, chemical and electrochemical exfoliation. As renewable energy and source utilization increase, including bioenergy from forest and woody residues, processed, among other methods, by pyrolysis treatment, it can be expected that biochar production will increase too. Thus, its useful applications, particularly in obtaining high-added-value products, need to be fully explored. This study aims at presenting a comprehensive analysis derived from experimental data, offering insights into the potential of biomass pyrolysis-derived biochar as a versatile precursor for the controlled synthesis of graphene and its derivatives. This approach comprehended the highest energy output and lowest negative environmental footprint, including the minimization of both toxic gas emissions during processing and heavy metals’ presence in the feedstock, toward obtaining biochar suitable to be modified, employing the Hummers and intercalation with persulfate salts methods, aiming at deriving graphene-like materials. Material characterization has revealed that besides morphology and structural features of the original wooden biomass, graphitized structures are present as well, which is proven clearly by Raman and XPS analyses. Electrochemical tests revealed higher conductivity in modified samples, implying their graphene-like nature.

## 1. Introduction

**Figure 1:**
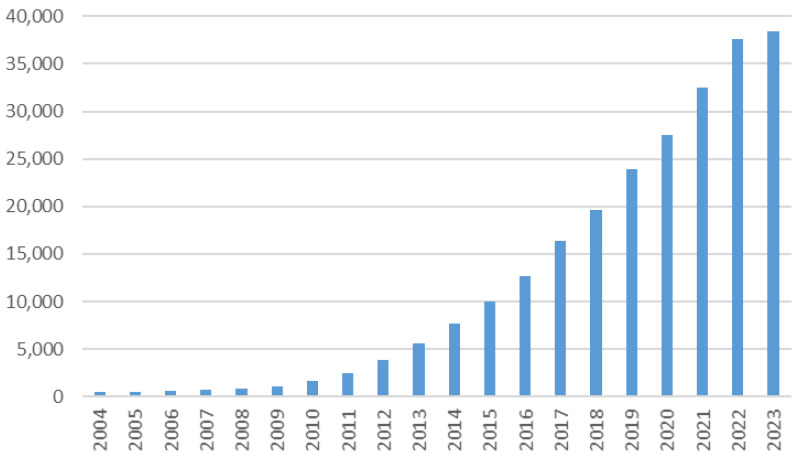
Increasing trend in graphene research from 2004 to 2023 (source: www.sciencedirect.com (14 November 2023), keyword: graphene).

Graphene, a two-dimensional carbon allotrope with a honeycomb structure, has emerged as a material of immense interest in diverse scientific domains owing to its unique and appealing properties such as high thermal and electrical conductivity, transparency, hardness, elasticity and flexibility [1,2,3]. Since single-layer graphene was first isolated in 2004 [4], scientific interest in this material and its application for various purposes such as electronics, medicine, composites and coatings, energy, catalysis, water purification and sensors has increased considerably and continues to grow, as depicted in Figure 1.

Various methods have been explored for the production of graphene, ranging from exfoliation of graphite to chemical vapor deposition and the reduction of graphene oxide [4,5,6,7]. The most widely studied raw material is graphite, which is a common precursor to graphene by mechanical, chemical and electrochemical exfoliation [8]. However, the graphite used for exfoliation may differ in quality, impurities and morphologies, which also affect the uniformity of the graphene product. Graphene can be produced from graphene oxide (GO), a hydrophilic, non-conductive carbon material with a graphene lattice structure with various oxygen-containing functionalities such as epoxide, carbonyl, carboxyl and hydroxyl groups. However, a large-scale adoption of the chemical reduction is hindered by long synthesis, as well as the costs and toxicity of effective reducing agents such as hydrazine [8]. Electrochemical reduction is fast, economically viable and environmentally friendly, as it does not involve the use of toxic reductants. However, the formation of defects often requires further processing of the electrochemically produced graphene.

The pursuit of sustainable and economically viable sources for graphene synthesis has led to the exploration of alternative precursors. The utilization of biomass pyrolysis-derived biochar as a precursor for graphene synthesis stands out as an environmentally friendly and economically viable approach. Renewable Energy Directive 2030 targets an increase in all areas of renewable sources utilization/production, including bioenergy from forest and woody residues processed, among other methods, by pyrolysis treatment [9,10]. Pyrolysis is a versatile technology for exploiting diversified feedstocks to produce a wide range of products, including biochar, bio-oil, and syngas via the decomposition of polymer chains in biomass macromolecules employing externally supplied heat under an inert atmosphere. It is considered as a thermochemical process for biomass processing, offering excellent control over process parameters, as well as resulting in low emissions of harmful gases [11]. As pyrolysis applications increase over the years, the biochar quantity generated via pyrolysis treatment will increase as well, and its useful application as a secondary raw material is highly demanded. Biochar, a carbonaceous material produced via the thermal decomposition of biomass in an oxygen-limited environment, has been extensively studied for its applications in soil amendment [12], carbon sequestration [13], and environmental remediation [14,15]. However, recent research has unveiled its potential as a precursor for the synthesis of graphene-based materials [5,16,17]. The inherent carbon-rich nature of biochar, along with its porous structure and heteroatom content, makes it a promising candidate for the controlled production of graphene and its derivatives.

This paper aims at exploring the utilization of biomass pyrolysis-derived biochar as a precursor for graphene synthesis, elucidating the mechanisms involved in the transformation from biochar to graphene-based materials. By understanding the transformation process from biochar to graphene and elucidating the impact of pyrolysis conditions chosen to minimize the negative environmental footprint through toxic gas emissions, starting with the selection of proper feedstock composition, then temperature conditions, and post-pyrolysis activation methods, this study seeks to uncover the optimal pathways for the conversion of biochar into high-quality graphene materials. The significance of this research lies in both the sustainable production of graphene materials and the valorization of biomass waste streams. The controlled synthesis of graphene from biochar not only offers an innovative approach towards graphene production but also contributes to the sustainable management of biomass waste streams.

## 2. Materials and Methods

### 2.1. Chemicals & Materials

**Figure 2:**
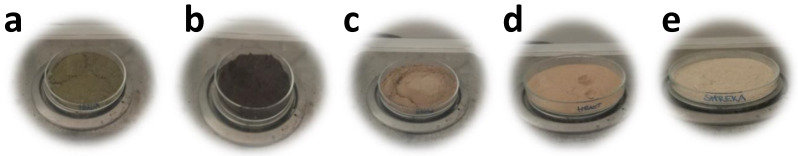
Biomass samples considered in the study: (**a**) grass (B-G); (**b**) coffee sludge (B-CS); (**c**) brewery sludge (B-BS); (**d**) wood chips, oak (B-WC/O) and (**e**) wood chips, spruce (B-WC/S).

The biomass samples considered in the study were grass (B-G) (collected at the location Pokupsko, Croatia, after seasonal cutting), coffee sludge (B-CS) (collected after preparation of coffee at the Department of Polymer Engineering and Organic Chemical Technology, Faculty of Chemical Engineering and Technology, University of Zagreb, Zagreb, Croatia, over a month period), brewery sludge (B-BS) (collected after beer preparation by Medvedgrad brewery, Zagreb, Croatia), and two types of wood chips, oak (B-WC/O) and spruce (B-WC/S) (collected at Forestry Department of Karlovac University of Applied Science, Karlovac, Croatia) (Figure 2).

The chemicals used in the study to modify the obtained biochar upon biomass pyrolysis into the graphene-like materials were: ammonium persulfate salt ((NH_4_)_2_S_2_O_8_, p.a., Kemika, Zagreb, Croatia), sodium carbonate (Na_2_CO_3_, p.a., Sigma Aldrich, Burlington, MA, USA), sulfuric acid (H_2_SO_4_, 97%, Lach-ner, Zagreb, Croatia), sodium nitrate (NaNO_3_, p.a., Kemika, Zagreb, Croatia), potassium permanganate (KMnO_4_, p.a., Kemika, Zagreb, Croatia), hydrogen peroxide (H_2_O_2_, 30%, Kemika, Zagreb, Croatia), and hydrochloric acid (HCl, 37%, Gram-mol, Zagreb, Croatia). Potassium chloride (KCl, p.a., Kemika, Zagreb, Croatia) was used as an electrolyte in electrochemical tests, while N-methyl pyrrolidone (C_5_H_9_NO, NMP, p.a., Sigma Aldrich, Burlington, MA, USA) and polyvinylidene fluoride (-(C_2_H_2_F_2_)n-, PVDF, p.a., Sigma Aldrich, Burlington, MA, USA) were used in specific capacity determination tests. Ethanol (CH_3_CH_2_OH, EtOH, 96%, Gram-mol, Zagreb, Croatia) was used for cleaning the pyrolysis unit between experimental cycles. MilliQ-water, obtained using a Direct-Q3 UV (Merck Millipore, Darmstadt, Germany) ultrapure water system, was used where necessary (washing, solution preparation, etc.).

### 2.2. Procedures

#### 2.2.1. Pyrolysis

**Figure 3:**
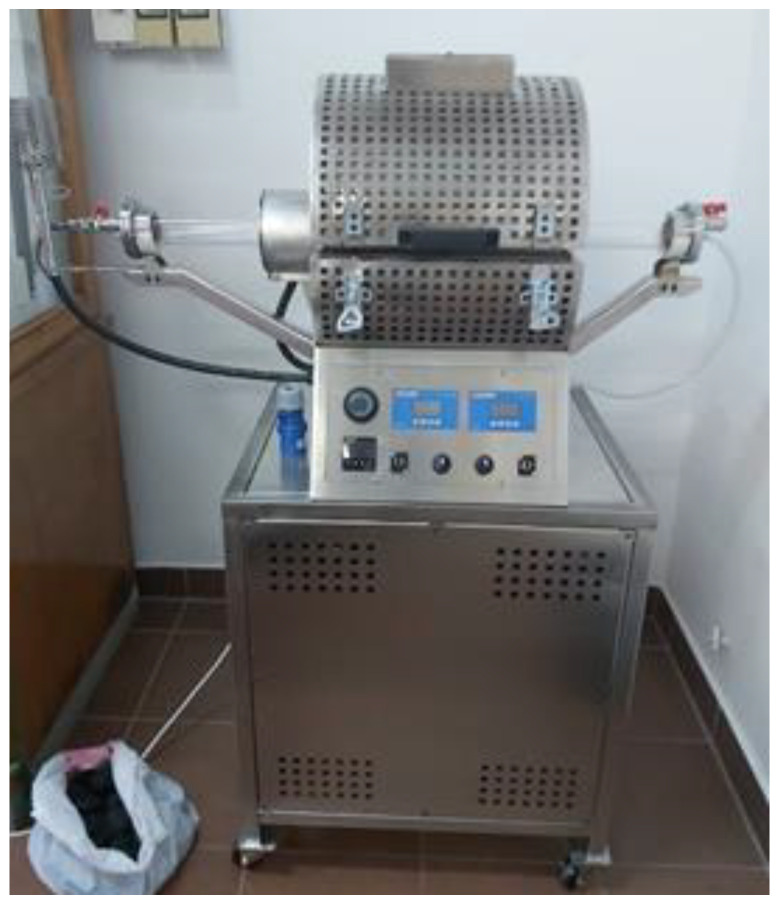
Custom-designed pyrolytic reactor used in the study for biomass processing.

The biomass samples were ground in a single-drum ball mill (Retsch, Haan, Germany) in 3 cycles of 40 s duration at 30 Hz. After the final grinding cycle, samples in a powder form were dried in a laboratory oven (UN-55, Memmert, Schwabach, Germany) at 105 °C up to the achievement of constant mass, and thereafter, were submitted to analysis, and thereafter, to pyrolysis processing (if selected). The pyrolysis of selected biomass samples on a small scale (when analysis of produced gaseous fraction was in focus) was performed using Pyroprobe 5000 (CDS Analytical, Oxford, PA, USA), employing temperatures of 400, 600 and 800 °C. The pyrolysis conditions were as follows: helium gas atmosphere with pressure stability and purge times of 20 and 10 s, respectively, heating rate of 10 °C/msec, overall duration of 10 min, while the biomass sample was 2 mg. For larger scale pyrolytic treatment of selected biomass samples (when biochar analysis and its further processing were in focus) a custom designed pyrolytic reactor (Estherm, Sveta Nedelja, Croatia) was employed (Figure 3). The same heating regime was employed as described above for small-scale process, while the biomass sample was increased to 10 g. The cover of reactor is stainless steel, while the heating unit is made of alumina-oxide isolation plate with embedded heaters. The furnace is divided into two separate zones with temperature regulation driven automatically including adjustment of heating temperature (max. 1150 °C), temperature ramp and speed. The reactor working space is made of quartz (V = 2 L). A vacuum pump was connected and running to ensure an inert atmosphere in the system, i.e., to prevent the oxidation of the material and to facilitate pyrolysis. The biomass (10 g) was introduced in the reactor space in a stainless steel sample boat, where biochar as a solid product remained after the pyrolysis process. Other products were either burned at the torch (gas fraction) or removed after the reaction from the quartz tube walls (oil fraction).

#### 2.2.2. Modification of the Obtained Biochar

The obtained biochar was modified using two methods: (i) intercalation with persulfate, and (ii) well-known Hummers method [18]. According to our past experience and the literature, both methods have been successfully employed to obtain graphene, but from graphite [18,19]. The first method considered homogenization of biochar and persulfate salt in a 1:5 ratio, then the addition of sulfuric acid in a quantity to completely cover solids. The mixture was vigorously mixed, transferred to an Erlenmayer flask which was covered with parafilm, and then was placed into a thermostated orbital shaker (120 rpm) for 24 h at 40 °C. It is necessary to make a small hole to serve as an exhaust for developing gases (CO_2_ and SO_2_). Afterwards, the sediment was separated from the suspension using vacuum filtration and was washed three times with water and then dried for 12 h at 40 °C in a laboratory oven (UN-55, Memmert, Schwabach, Germany). The final stage considered treatment in a muffle furnace (LHT 08/18/P470, Nabertherm, Lilienthal, Germany) for 15 s, and then cooling the resulting material at the room temperature. The second method used for modifying obtained biochar was based on the Hummers method [18] and included treating 3 g of biochar with 69 mL of H_2_SO_4_ and 1.5 g of NaNO_3_ in an ice bath. Then, 9 g of KMnO_4_ was added to the mixture, taking care not to rise the temperature significantly. After 1 h of stirring, demineralized water was slowly added until the temperature reached near boiling. The mixture was then allowed to cool and was subsequently diluted with 1 L of deionized water. H_2_O_2_ was then added to the diluted solution, until the cessation of the observed effervescence. The mixture was centrifuged, and the whitish precipitate was discarded, while the brownish floating product was rinsed with 5% HCl. The residual acid was washed until the suspension became nearly pH neutral. The suspension was dialyzed for 14 days with a Spectrum chemical Spectra/Por membrane (Thermo Scientific, Waltham, MA, USA) in ultrapure water. Water from the obtained suspension was removed in a vacuum at 35 °C.

### 2.3. Analyses

The biomass materials were characterized prior to pyrolysis for (i) calorific value using IKA C4000 calorimeter (IKA-Werke GmbH & Co. KG, Staufen, Germany) employing standardized methodology (HRN EN 14918:2009) [20]; (ii) elemental analysis using Vario Macro cube CHNS elemental analyzer (Elementar, Langenselbold, Germany) employing Pregl-Dumas method and standards HRN ISO 13878:2004 [21] HRN ISO 10694:2004 [22] HRN ISO 15178:2005 [23]and HRN EN 15104:2011 [24] for nitrogen (N), carbon (C), sulphur (S) and hydrogen (H) determination; and (iii) heavy metals content analysis using ICP-MS (ELAN DRC-e, Perkin Elmer, Woodbridge, ON, Canada), with microwave digestion (Advanced Microwave Labstation, ETHOS 1600, Milestone, Italy) for sample preparation.

The gases produced from the pyrolysis of biomass were analyzed using a model GCMS-QP2020 NX MS (Shimadzu, Kyoto, Japan) coupled to a GC-2020 gas chromatograph (Shimadzu, Kyoto, Japan). A 30 m × 0.530 mm DB-WAX UI column (Agilent Technologies, Santa Clara, CA, USA) was employed for the analysis. Helium was used as the carrier gas at a constant linear velocity of 36.3 cm min^−1^, with a split ratio of 1:100. The injector temperature was set to 200 °C, while the GC oven temperature was set initially at 120 °C for 5 min, and then ramped at a rate of 25 °C min^−1^ to 245 °C. Then, the column was held at 245 °C for a further 5 min, after which the oven temperature was ramped at a rate of 3.5 °C min^−1^ to 285 °C for 18 min.

The obtained biochar was characterized prior to and after the modification for morphology, structure and surface properties. Hence, scanning electron microscopy (SEM) using Ultra Plus SEM (Zeiss, Jena, Germany), along with energy dispersive spectroscopy spectra (EDS) using X-max silicon drift detector (Oxford, UK), were employed. The samples were loaded on a graphite adhesive tape, without vapor phase deposition pretreatment. X-ray Photoelectron Spectroscopy (XPS) measurements were performed using a PHI VersaProbe III (Version AD) (PHI, Chanhassen, MN, USA) equipped with a hemispherical analyzer and a monochromatic Al Kα X-ray source. Survey spectra were measured using a pass energy of 224 eV and step of 0.8 eV, while Fe *2p* core level spectra were measured at pass energy of 27 eV and step of 0.1 eV. The data were acquired using the ESCApe 1.4 software. Fitting of C1s core level spectra was performed using CasaXPS software 2.3.15. Raman spectroscopy were measured using an Alpha300 (Witec, Ulm, Germany) equipped with a microscope and attached atomic force microscope (AFM). Excitation source wavelength was set to 532 nm, while integration time was set to 5 s with an average of 20 scans taken.

Cyclic voltammetry measurements were performed in 0.1 mol dm^−3^ KCl solution using a potentiostat/galvanostat (PalmSens4, PalmSensBV, Houten, The Netherlands), equipped with a standard three-electrode system: a carbon-paste electrode with (modified) biochar sample was used as working electrode, a saturated calomel electrode was used as a reference electrode and a Pt foil (*A* = 0.5 cm^2^) as counter electrode. The experiment was carried out in a potential range from −0.8 to 0.8 V, with a scan rate of 50 mV s^−1^. The working electrode was prepared using a cylindrical holder filled with carbon paste, which served as a holder for (modified) biochar samples. In the next step, it was polished using a sheet of paper until a shiny surface was obtained. After examination of the prepared electrode, different samples were carefully attached to the carbon paste electrode.

Specific capacity was determined using a glassy carbon working electrode with deposited sample of (modified) biochar in a form of suspension. The suspension was prepared by dissolving 10 mg of biochar sample in 1 mL of NMP, and then 1 mg of PVDF binder was added. The suspension was homogenized using ultrasound. In order to prepare glassy carbon covered by different samples, 10 μL of sample suspension was drop-casted on clean GC disc electrode (A = 0.07 cm^2^) and dried in vacuum oven for 24 h. Specific capacitance, Cs (F g^−1^), was calculated from the cyclic voltamogramm curves according to Equation (1) [25]:

(1) $$C_{s}=\frac{\int_{E1}^{E2}IdE}{2m\vartheta (E_{1}-E_{2})}$$

where C_s_ is the specific capacitance (F g^–1^), I is the current, A, E_1_ is the initial potential (V), E_2_ is the final potential (V), n is the scan rate (V s^–1^), m is the mass of tested material.

The electronic resistivity and conductivity was measured using pressed (modified) biochar samples and 34461A Digital Multimeter (Keysight, Santa Rosa, CA, USA) with four-point probe.

Electrical conductivity was determined according to Equations (2) and (3) [26]:

(2) $$\rho =\frac{\pi dR}{ln2}$$

(3) $$\kappa =\frac{1}{\rho }$$

where ρ is resistivity (Ω cm), R is resistance (Ω), d is thickness of the sample (m), and κ conductivity (S cm^–1^).

## 3. Results

### 3.1. Analysis of Biomass and Selection of for Pyrolysis

The selection of the biomass type to be submitted for pyrolysis was performed based on several criteria. Hence, the calorific values, elemental analysis and heavy metals content were determined, and the samples with the highest calorific values and lowest heavy metal content were selected. In Table 1, the results of the determined calorific values and performed elemental analysis are summarized, while the content of a vast array of heavy metals detected in the biomass samples is provided in Table S1 (Supplementary Materials). As can be seen, the highest calorific value of 22.25 MJ kg^−1^ is determined for the B-BS sample (brewery sludge), which was followed with three biomass samples with similar calorific values: B-CS, B-WC/S and B-WC/O (in decreasing order), while the far lowest value was obtained for the B-G sample (grass). A similar order can be seen in C and H content, which can be closely correlated with the calorific value; the higher overall C and H contents, the higher the heating value index of biomass too [27]. It should be also noted that the heating value of H is ~7 times higher than that of C; thus, more H per C leads to more energy. The values of the effective hydrogen-to-carbon atomic ratio (H/C_eff_), calculated using Equation (4) [28]:

(4) $$\frac{H}{C_{eff}}=\frac{moles\;of\;H-(2*moles\;of\;O)}{moles\;of\;C}$$

are presented in Table 1. Over this parameter, the pyrolysis yield generating oil and coke (i.e., biochar in our case) products can be estimated. Hence, when the H/C_eff_ value of a biomass is <1, meaning that the biomass is hydrogen deficient, biochar content would be higher, and vice versa in favor of oil.

As can be seen, the sludgy biomass samples possess a H/C_eff_ higher than 1, which is in accordance with their rather high calorific values. B-WC/O has a H/C_eff_ value close to 1 (0.98), which is beneficial regarding the goal of the study: to valorize the biochar produced during pyrolysis.

**Figure 4:**
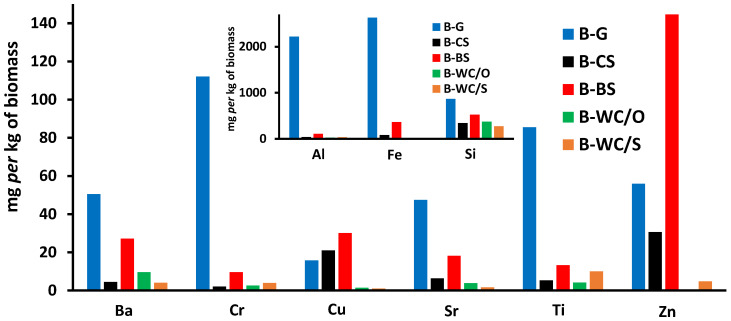
Content of selected heavy metals: Ba, Cr, Cu, Sr, Ti and Zn in studied biomass samples. Inset figure shows Al, Fe and Si content in biomass samples.

In addition, the content of heavy metals detected in the biomass samples speaks in favor of such a conclusion. As can be seen from Figure 4, it shows the selected heavy metals either present at a higher concentration such as Al, Fe and Si, or those with documented and potential adverse effects to environment and humans such as Cr, Ba, Sr, Zn, Cu and Ti. It is known that the presence of heavy metals as impurities in graphite-like materials is not beneficial for their transformation to graphene-like materials [29]; although, the presence of certain transition metals was shown to be beneficial when biochar based-materials were used as adsorbents/catalysts for water and soil remediation [14,30]. As can be seen from Figure 4, the B-G sample (i.e., grass) possesses the highest values of detected heavy metals by far (full list is provided in Table S1, Supplementary Materials); the exceptions are Cu (both sludgy biomass samples B-CS and B-BS possess somewhat higher values) and Zn (B-BS sample has ~3 times higher content). On the other hand, woody biomass samples, B-WC/O and B-WC/S, have very low concentrations of heavy metals (or even none for some elements; Table S1, Supplementary Materials). Hence, those are much better candidates to be pyrolyzed in order to valorize the obtained biochar as a potential precursor for graphene-like materials. According to the selection criteria, as well as the wide-spread oak population and application in the wood processing industry (furniture, parquet, etc.) in Croatia, and as such large quantities of chips are to be potentially processed in commercial pyrolysis units, the B-WC/O sample was selected for further study, i.e., it was submitted for pyrolysis.

### 3.2. Pyrolysis of B-WC/O Biomass Sample

According to the above-mentioned selection criteria and presented results, the biomass sample B-WC/O was submitted to pyrolysis, employing three different temperatures: 400, 600 and 800 °C. 

**Figure 5:**
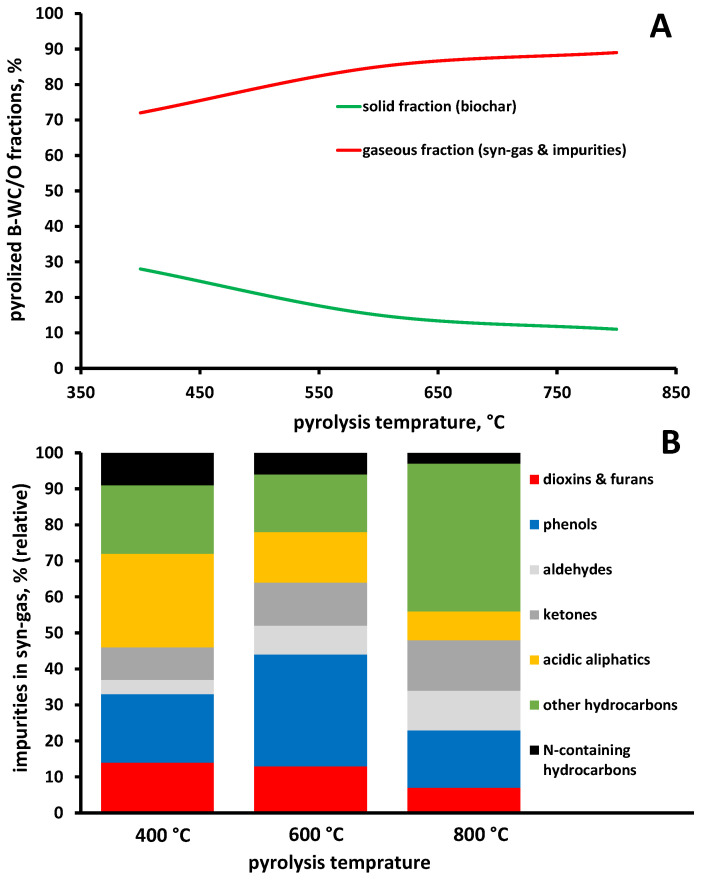
The ratio of solid and gaseous fractions produced during B-WC/O sample pyrolysis at different temperatures (**A**), and relative content of impurities present in gaseous fraction at different pyrolysis temperatures (**B**).

The generation of pyrolysis products was monitored, both quantitatively and qualitatively. The quantitative analysis included determination of pyrolysis product fractions: solids (biochar), liquids (oil) and gaseous (syn-gas). Hence, the content of solids (i.e., biochar) was determined gravimetrically after the pyrolysis process, while it should be noted that liquid fraction production was not noticed at any temperature in significant extents (just small smudgy leftovers on the reactor walls, i.e., quartz tube). Thus, it is considered that rest of biomass was transformed mainly into gaseous products. Accordingly, as showed in Figure 5A, the elevation of pyrolysis temperature caused a lowering of solids content, which was followed by a proportional increase in gaseous products. Such findings are in accordance with the literature data [31]. The obtained gaseous fraction was qualitatively and quantitatively analyzed for the content of impurities, particularly focusing on those generating serious adverse effects to the environment and humans (dioxins and furans), while solids were thoroughly analyzed and valorized in the following section in order to inspect their application as precursors for graphene-like materials. Hence, the gaseous fraction produced during pyrolysis was analyzed using GC-MS. It should be noted that quantitative analysis of syn-gas main constituents such as H_2_, CO, CO_2_ and CH_4_ was not performed due to methodology limitations. On the other hand, thorough inspection of potentially harmful impurities formed in the gaseous fraction was performed; the chromatograms containing all produced gas phase constituents (except main syn-gas constituents stated above) is provided in Figure S1 (Supplementary Materials). Generally, all three chromatograms looks similar, and even the groups of impurity products are similar. Figure 5B shows the impurities summarized into several groups regarding their structure: (i) dioxins and furans, (ii) phenols, (iii) aldehydes, (iv) ketones, (v) acidic aliphatics, (vi) other hydrocarbons and (vii) N-containing hydrocarbons. The main representatives detected (and identified using NIST base compatibility ≥ 90%) of those groups formed at different temperatures are showed in Table 2. As can be seen, among impurities, both aliphatic and aromatic compounds are present, even among the “other hydrocarbons” group which also included lower C-containing aliphatic compounds without heteroatoms (e.g., propane, propene, butanes, etc.).

**Figure 6:**
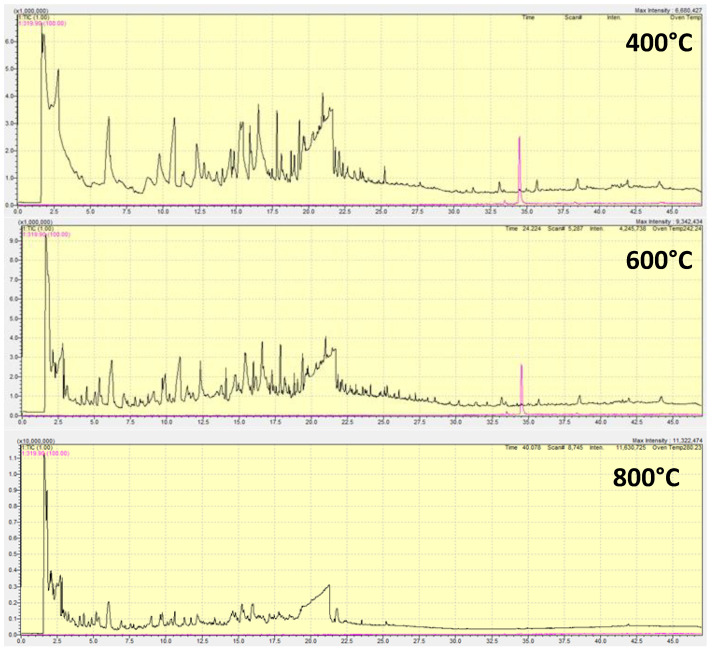
The generation of hazardous dioxin product, 2,3,7,8-TCDD, at different pyrolysis temperatures.

It should be noted that an increase in pyrolysis temperature caused different distributions of impurities formed within the gaseous phase. Hence, dioxins and furans were double lowered when the temperature was increased from 400 to 800 °C. The aldehydes and ketones somewhat increased with the temperature elevation, while aliphatic acids and N-containing hydrocarbons decreased during that temperature interval increase. The most significant increase can be observed in the “other hydrocarbons” group, probably due to the formation of methane precursors (lower C-containing hydrocarbons without heteroatoms), while oxygen would be combined with carbon into CO and eventually into CO_2_. It is interesting that although nitrogen was present in a negligible amount in the pristine B-WC/O sample (<0.1%), N-containing impurities were formed. However, the impurities of emerging concern to be monitored, and if possible, minimized (or completely diminished) in pyrolysis gas are those pertaining to the “dioxins and furans” group. In Table 3, selected hazardous compounds pertaining to the “dioxins and furans” group and formed during pyrolysis of the B-WC/O sample at different temperatures are summarized, along with their mass weights (based on the literature data [32] and quantities, expressed in “absolute” values (estimated over area under the pertaining peak) and relative values (to the content formed at the lowest pyrolysis temperature, 400 °C). As can be seen, in most cases, the highest amounts of selected dioxins and furans were formed at the lowest temperature, while at the highest temperature of pyrolysis studied, most of these hazardous compounds were not event formed. That is clearly depicted in Figure 6, presenting the chromatograms for the overall gaseous phase formed, along with that of 2,3,7,8-tetrachlorodibenzodioxin (2,3,7,8-TCDD) (present with pink color). As can be seen, at 800 °C, that compound is not formed at all. It should be noted that selected 2,3,7,8-TCDD is among the most potent compounds (congener) of the dioxin series, and was identified worldwide, even as consequences of war activities (Agent Orange sprayed in Vietnam in the 1970s was contaminated by 2,3,7,8-TCDD [33]) and terrible accidents too (Seveso; [34]). Hence, the biochar for further processing, i.e., for the valorization to test its ability to serve as a precursor of graphene-like materials, was used from the pyrolysis treatment of B-WC/O at 800 °C.

### 3.3. Valorization of Biochar and Its Graphene-like Derivates

**Figure 7:**
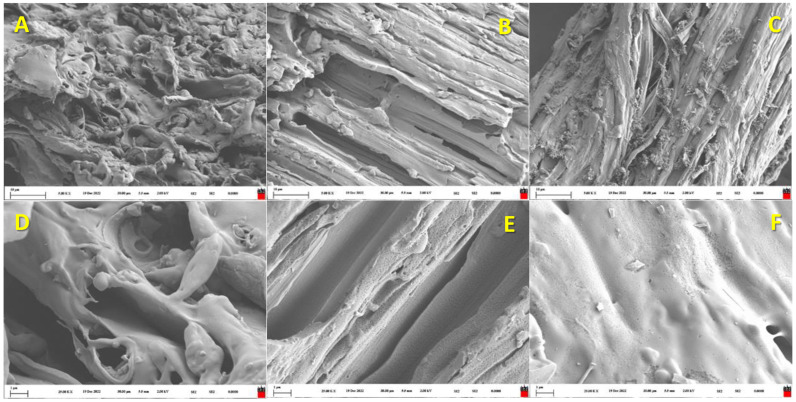
The SEM images of pristine biochar (**A**,**D**) and its graphene-like derivatives obtained by Hummers (**B**,**E**) and intercalation with persulfate (**C**,**F**): top row has lower magnification, while bottom row is made with higher magnification.

The obtained biochar from pyrolysis of the B-WC/O sample was collected after the treatment, and thereafter, its modifications by the Hummmers method [18] and intercalation with persulfate salts [19,35] were attempted in order to obtain a graphene-like material. In order to inspect the structural and morphological properties, as well electronic conductivity, all three materials, pristine biochar and those modified by two types of aforementioned methods, were thoroughly analyzed. In Figure 7, SEM images of all three materials are shown. As can be seen in the three micrographs of the studied samples of biochar, pristine and the two modified materials, each has different surface structures. However, in all cases, the general structure resembles the morphology of the initial wooden biomass, i.e., the xylem and phloem structures are discernable, despite the pyrolysis treatment and performed modifications in samples Figure 7B,C,E,F. In the case of pristine biochar (Figure 7A,D), amorphous structures can be observed, which are probably incomplete pyrolyzed structures. In the sample of biochar modified by the Hummers method (Figure 7B,E), numerous pinholes can be seen all over the surface of the material, suggesting that selective oxidation of the plant structure by H_2_SO_4_ and KMnO_4_ took place. The individual layer of the biomass can be also observed. The sample prepared by intercalation with persulfate salts (Figure 7C,F) is more similar to pristine biochar than to the sample modified by the Hummers method. Xylem parts are still visible due to partial hydrolysis; however, layers are formed at some places. The EDX analysis showed that pristine biochar and the one modified by intercalation with persulfate salts contained impurities detected in the biomass sample prior to the pyrolysis process at a higher extent: magnesium (Mg), calcium (Ca) and chlorine (Cl). The sample modified by Hummers did not show traces of those elements; actually, the EDX mapping showed just a high intensity of C and O atoms (Figure S2, Supplementary Material). Hence, it can be concluded that mineral impurities are presumably removed by the Hummers process.

**Figure 8:**
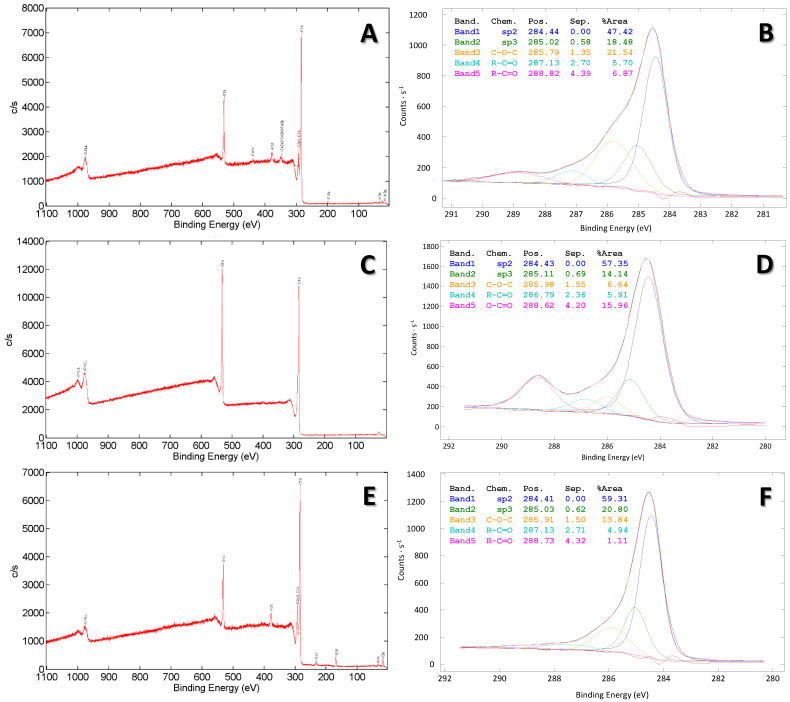
The XPS survey spectra (**left column**) and C1s core-level spectrum (**right column**) of pristine biochar (**A**,**B**) and its graphene-like derivatives obtained by Hummers (**C**,**D**) and intercalation with persulfate (**E**,**F**).

The next task was to inspect the surface properties of the studied samples, employing XPS analysis. As can be seen from the survey spectra (Figure 8, left column), the biochar modified by the Hummers method has the simplest elemental composition, only carbon and oxygen (Figure 8C). On the other hand, the other two samples possess other elements in their surface structure. Hence, pristine biochar has some remained impurities such as K, Ca and Cl (Figure 8A), as also observed by EDX analysis. However, some of those are not detected in a surface inspection of the biochar modified by intercalation with persulfates (Figure 8E), although they were present in the sample during EDX analysis. XPS analysis showed the presence of sulfur and potassium, which are more likely to be from the remaining persulfate salt than from the pristine biomass. Moreover, sulfur is present in the B-WC/O sample prior to pyrolysis treatment in traces (Table 1). As can be seen from the right column in Figure 8, in all three samples, C1′s core spectra was convoluted, with five sub-bands presenting main hybridization states and specific bonds; their contributions are presented in Table 4. The prevailing carbon form is in an *sp^2^* hybridized bond, with a maximum of 284.4 eV, which is characterized with delocalized π-electrons. In the case of pristine biochar, the contributing form is C-O-C/C-OH, representing epoxy and hydroxyl bonds with a bonding energy of 285.89 eV, which was followed by an *sp^3^* hybridized bond with a bonding energy of 285.02 eV. On the other hand, in the case of the biochar modified by Hummers, the second contributing form is O-C=O/CO3, representing carbonyl bonds, with a maximum of 288.62 eV, which was followed by the *sp^3^* hybridized bond. In the biochar sample modified by intercalation with persulfate, *sp^3^* hybridized is the second most contributing bond, followed by C-O-C/C-OH [36].

**Figure 9:**
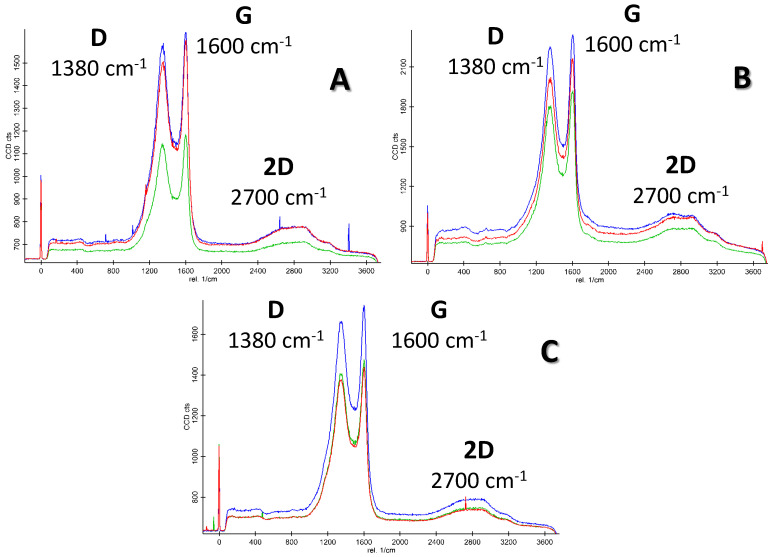
Raman spectra of pristine biochar (**A**) and its graphene-like derivatives obtained by Hummers (**B**) and intercalation with persulfate (**C**) (laser induction of 1.4 mW).

The Raman spectra (excited with green laser light at 532 nm) of the three studied biochar samples are showed in Figure 9. As can be seen, three type of peaks can be observed in all three cases: D, G and 2D appearing in graphitic structures at intervals 1200–1500 cm^−1^, 1500–1800 cm^−1^ and 2700 cm^−1^, respectively [37]. Hence, the D peak appears at 1380 cm^−1^, suggesting the presence of defects attached to the basal plane of a graphite crystalline structure. The G peak appears at 1600 cm^−1^ in our samples, and is due to vibration stretching of C-C bonds within an aromatic ring, which is characteristic of *sp^2^* hybridized structures [38,39,40], proving the graphitic nature of the studied biochar samples. Additionally, the appearance of the 2D peak at 2700 cm^−1^ also confirms the presence of a graphitic phase in our samples [37]. It should be noted that the Raman spectra of the biochar samples obtained in this study are very similar to those in the literature sources studying biochar materials [41].

**Figure 10:**
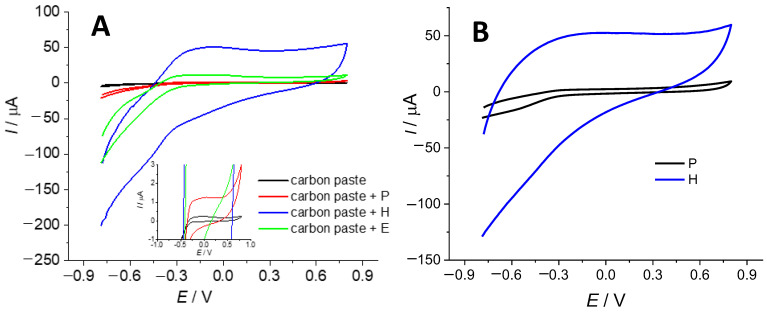
Cyclic voltammogram of carbon paste electrode covered by biochar samples (**A**) and glassy carbon electrode covered by biochar samples (**B**).

Figure 10 represents the cyclic voltammetry responses of the three different samples: pristine biochar and those modified by Hummers and intercalation by persulfate salts. The obtained current values correspond to electrochemical double layer charging at the biochar/solution interface. The first measurement was carried out for bare support (carbon paste electrode, Figure 10A) from where it is evident that higher current values are obtained for the electrode with the modified samples compared to those obtained by the carbon-paste electrodes. This result indicates good conductivity of biochar that was expectable for the carbon-containing *sp^2^* structure. Namely, good conductivity is a necessary condition for double layer charging/discharging. The highest current value was registered for the electrode containing the biochar modified by Hummers.

A similar experiment was carried out by applying pristine biochar and biochar modified by Hummers to a glassy carbon electrode. In this case, the specific capacitance was calculated according to Equation (1) (Table 5). It is evident that a higher specific capacitance value, Cs, was recorded for the electrode containing the biochar modified by Hummers than that for the electrode containing pristine biochar. This result is surprising considering that the best electrical conductivity was recorded for pristine biochar (Table 6). However, there are two explanations for such behavior: (a) the higher number of hydrophilic groups within the structure of biochar modified by Hummers improves its wettability and increases the suitability of the surface for double layer charging and (b) the biochar modified by Hummers contains a lower amount of impurities that do not participate in double layer charging.

## 4. Conclusions

In order to understand the transformation process from biochar to graphene and to elucidate the impact of pyrolysis conditions chosen to minimize a negative environmental footprint through toxic gas emissions, the optimal pathways to uncover the conversion of biochar into high-quality graphene materials were explored. Hence, the selection of proper biomass started by exploring its composition through elemental analysis, including both energy valuable parameters, estimated using calorific value and hydrogen-to-carbon atomic ratio, and those raising environmental concern, estimated by the detection of heavy metals content. Hence, among several biomass sources, namely cut grass (B-G), coffee sludge (B-CS), brewery sludge (B-BS), spruce (B-WC/S) and oak (B-WC/O) woodchips, the latter was chosen as a feedstock for pyrolysis under a vacuum in order to obtain biochar due to favorable energetic values (calorific value of 19.39 (±0.18) MJ kg^−1^ and H/C_eff_ of 0.98) and the lowest amount of heavy metals among the studied biomass candidates. At higher temperatures, namely 800 °C, no dioxins were detected in the gaseous pyrolysis products; thus, this temperature was selected for biochar production for further investigation. The resulting biochar was subsequently treated using the Hummers method and intercalation with persulfate salts, aiming at deriving graphene-like materials. Material characterization has revealed that, despite pyrolysis and these chemical post-treatments, the morphology and structural features of the original wooden biomass were retained in the biochar but to a lesser extent in the modified samples. Furthermore, Raman spectra confirmed the graphitic nature of the carbon in both the pristine and modified biochar samples, which is in agreement with the literature. Electrochemical tests exhibited the highest conductivity in samples modified by the Hummers method, indicating their close-to-graphene-like structure. However, it has to be concluded that a higher pyrolysis temperature would probably lead to more graphitized biomass to such an extent as to be favorable for the production of graphene-like materials; although, the obtained modified materials in our study showed some effects typical of graphene-like materials. 

## Figures and Tables

**Table 1:** Determined calorific values and elemental analysis of studied biomass samples.

	B-G	B-CS	B-BS	B-WC/O	B-WC/S
Calorific values (MJ kg^−1^)					
	17.04 (±0.11)	19.98 (±0.10)	22.25 (±0.14)	19.39 (±0.18)	19.76 (±0.16)
Elemental analysis					
C, %	46.7	55.9	53.0	52.1	51.3
H, %	8.5	10.5	10.0	9.1	8.6
O, %	40.8	30.8	33.3	38.8	40.0
N, %	3.2	2.5	3.3	<0.1	<0.1
S, %	0.2	0.2	0.2	<0.1	<0.1
effective hydrogen-to-carbon atomic ratio (H/C_eff_)					
	0.87	1.43	1.32	0.98	0.84

**Table 2:** The main representatives of impurities detected, classified under structure-based groups.

Group Name	Compound Name	Retention Time
Dioxins and Furans	2,5-dimethylfuran	3.117
	2,3-dihydro-5,6-dimethyl-1,4-dioxine	14.703
Phenols	2-methoxyphenol	12.329
	4-ethyl-2-methoxyhenol	15.418
Aldehydes	hydroxyacetaldehyde	2.319
	pentadecanal	18.443
Ketones	1-penten-3-one	4.107
	1,2-cyclopentanedione	9.112
Aliphatic acids	formic acid	2.127
	acetic acid	2.814
Other hydrocarbons	3,5-dimethoxy-4-hydroxytoluene	17.866
	triacontane	22.816
N-containing hydrocarbons	2,4-diaminopyrimidine	10.193
	2,2-diethyl-3-methyl-oxazolidine	10.908

**Table 3:** The selected dioxins and furans formed during the pyrolysis of B-WC/O at different temperatures (with **bold red** font are marked compounds where increase comparing to the lowest used temperature was recorded at elevated conditions).

Mw	Compound Name	400 °C	600 °C		800 °C	
		Area	Area	%	Area	%
96	furfural	22,383,206	13,850,372	61.9	7,599,849	34.0
120.15	phthalan	321,587	300,774	93.5	461,073	**143.4**
98.1	2-furanmethanol	10,442,656	6,337,734	60.7	3,497,492	33.5
319.9	2,3,7,8-TCDD	169,125	177,125	**104.7**	/	/
339.9	1,2,3,7,8-penta CDD	326,782	263,358	80.6	/	/
355.9	2,3,4,7,8-penta CDF	346,348	262,520	75.8	/	/
301.9	^13^C-PCB (169, 52, 80, 81)	1,149,042	1,216,012	**105.8**	6425	0.6
289.9	PCB (52, 81, 77, 153, 138, 167, 156, 157)	69,184	74,981	**108.4**	/	/
325.9	PCB (101, 123, 118, 114, 105) ^13^C_6_-1234-TCDD	306,252	276,334	90.2	/	/
303.9	^13^C-12378-PeCDD	164,730	157,342	95.5	/	/
200	^13^C-PCB 28	82,622	73,534	89.0	34,906	42.3
303.8	^13^C-PCB (153, 138, 167, 156)	161,931	131,856	81.4	/	/
285.9	^13^C-23478-PeCDF ^13^C-12378-PeCDF	152,855	213,608	**139.8**	/	/

**Table 4:** Relative contributions of carbon forms to C1s (XPS analysis) and intensity ratio (Raman analysis).

Biochar Type	*sp* ^2^	*sp* ^3^	C-O-C/C-OH	R-C=O/O-C-O	R-C=O/O-C-O	O-C=O/CO3	*I*_D_/*I*_G_
pristine	47.42	18.48	21.54	5.70	6.87	/	0.95
Modified by Hummers	57.35	14.14	6.64	5.91	/	15.96	0.89
Modified by persulfate	59.31	20.80	13.84	4.94	1.11	/	0.95

**Table 5:** Specific capacitance values for the pristine biochar and biochar modified by Hummers.

Biochar Type	Pristine	Modified by Hummers
Cs [F/g]	0.55	7.13	

**Table 6:** Electrical conductivity of different biochar types.

Biochar Type	Pristine	Modified by Hummers	Modified by Persulfate
κ [S cm^−1^]	2.81	0.45	0.55

## Data Availability

The datasets collected and analyzed in this work are available from the corresponding author upon reasonable written request.

## Supplementary Materials

The following supporting information can be downloaded at: https://www.mdpi.com/article/10.3390/ma16247658/s1, Figure S1: Chromatograms showing all produced gas phase constituents during pyrolysis of sample B-WC/O at three different temperatures: 400, 600 and 800 °C; Figure S2: EDX mapping of pristine (A) and modified biochar samples (B, Hummers, and C, persulfate intercalation); Figure S3: FTIR spectra of pristine and biochar samples modified by Hummers and persulfate intercalation; Table S1: Content of all detected heavy metals within studied biomass samples (mg per kg of biomass sample).
