# Supplementary material for: Biomass Pyrolysis-Derived Biochar: A Versatile Precursor for Graphene Synthesis
Source: Materials (Basel). 2023 Dec 15;16(24):7658. doi: 10.3390/ma16247658 (PMC10744795; doi:10.3390/ma16247658)
Supplement: Supplementary file 1 [file materials-16-07658-s001.zip › materials-2747801-supplementary.pdf]

Supplementary material

Biomass Pyrolysis-Derived Biochar: A Versatile Precursor for Graphene Synthesis

Karla Plenča [1], Sara Cvetnić [2], Helena Prskalo [2], Marin Kovačić [1], Matija Cvetnić [1], Hrvoje Kušić [1,3,*], Zvonimir Matusinović [2], Marijana Kraljić Roković [1], Boštjan Genorio [4], Urška Lavrenčič Štangar [4], Ana Ginčarić Božić [1**]

[1]Faculty of Chemical Engineering and Technology, University of Zagreb, Marulićev trg 19, 10000 Zagreb, Croatia; kplenca@fkit.hr (K.P.); mkovacic@fkit.hr (M.K.); mcvetnic@fkit.hr (M.C.); hkusic@fkit.hr (H.K.); mkralj@fkit.hr (M.K.R.), abozic@fkit.hr (A.L.B.)

[2]Karlovac University of Applied Sciences, Trg J.J. Strossmayera 9, Karlovac 47000, Croatia; sara.cvetkovic789@gmail.com (S.C.); prskalohelena5@gmail.com (H.P.); zvonimir.matusinovic@vuka.hr (Z.M.)

[3]Department for Packaging, Recycling and Environmental Protection, University North, Trg dr. Žarka Dolinara 1, Koprivnica 48000, Croatia; hkusic@fkit.hr (H.K.)

[4]University of Ljubljana, Faculty of Chemistry and Chemical Technology, Večna pot 113, SI-1000 Ljubljana, Slovenia; bostjan.genorio@fkkt.uni-lj.si (B.G.); Urska.Lavrencic.Stangar@fkkt.uni-lj.si (U.L.S.)

* Correspondence: hkusic@fkit.hr (H.K.); abozic@fkit.hr (A.L.B.)

Table S1. Content of all detected heavy metals within studied biomass samples (mg per kg of biomass sample)

	**B-G**	**B-CS**	**B-BS**	**B-WC/O**	**B-WC/S**
Ag	<0.001	<0.001	<0.001	<0.001	<0.001
**Al**	2.2×10^{3}	37.7	105.9	23.9	35.5
As	1.213	0.004	0.037	0.030	0.005
Au	<0.001	<0.001	<0.001	<0.001	<0.001
B	<0.001	<0.001	<0.001	<0.001	<0.001
Ba	50.5	4.49	27.1	9.55	3.95
Be	0.123	<0.001	0.000	0.002	0.003
Cd	0.149	0.001	0.084	0.017	0.149
Ce	<0.001	<0.001	<0.001	<0.001	<0.001
Co	1.504	0.210	0.092	0.044	0.036
Cr	112.1	2.09	9.59	2.53	3.94
Cs	0.628	0.009	0.013	0.003	0.024
Cu	15.72	20.98	30.12	1.39	0.99
Dy	0.325	0.002	0.010	0.003	0.001
Er	0.175	0.001	0.005	0.001	0.000
Eu	<0.001	<0.001	<0.001	<0.001	<0.001
**Fe**	2.6×10^{3}	80.2	0.4×10^{3}	15.7	21.2
**Si**	8.7×10^{2}	3.4×10^{2}	5.2×10^{2}	3.7×10^{2}	2.7×10^{2}
Sm	<0.001	<0.001	<0.001	<0.001	<0.001
Sn	0.647	0.219	0.490	0.455	0.321
Sr	47.4	6.34	18.1	3.78	1.64
Ta	0.009	<0.001	0.016	0.003	<0.001
Te	<0.001	<0.001	<0.001	<0.001	<0.001
Th	0.846	<0.001	<0.001	<0.001	<0.001
Ti	85.5	5.30	13.2	4.09	10.0
Tl	<0.001	<0.001	<0.001	<0.001	<0.001
Tm	<0.001	<0.001	<0.001	<0.001	<0.001
U	0.150	0.002	0.028	<0.001	<0.001
V	5.83	<0.001	0.76	0.05	0.01
W	0.075	<0.001	<0.001	<0.001	<0.001
Yb	<0.001	<0.001	<0.001	<0.001	<0.001
**Zn**	55.9	30.6	144.6	0.30	4.74
Zr	0.670	0.068	0.160	0.046	0.038

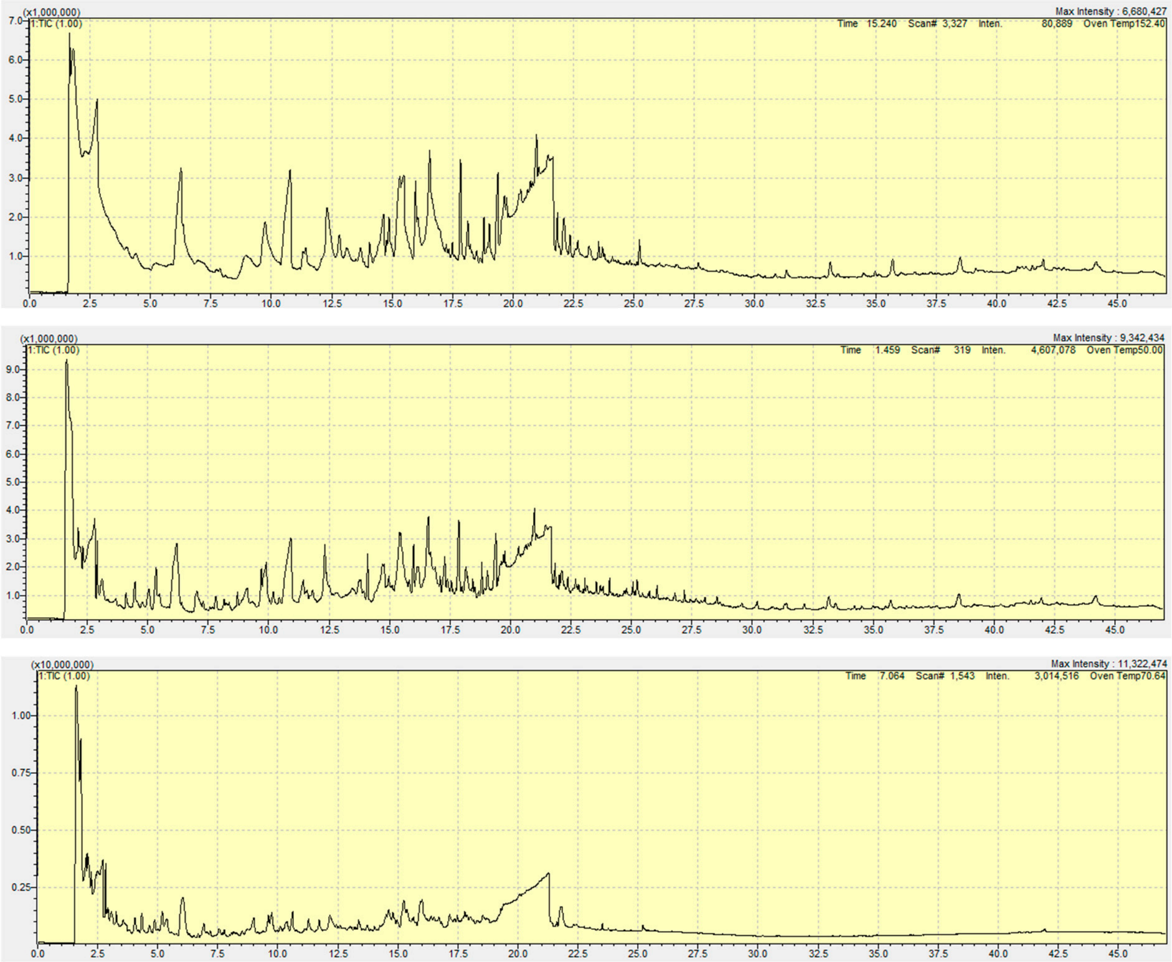

Figure S1. Chromatograms showing all produced gas phase constituents during pyrolysis of sample B-WC/O at three different temperatures: 400, 600 and 800 °C

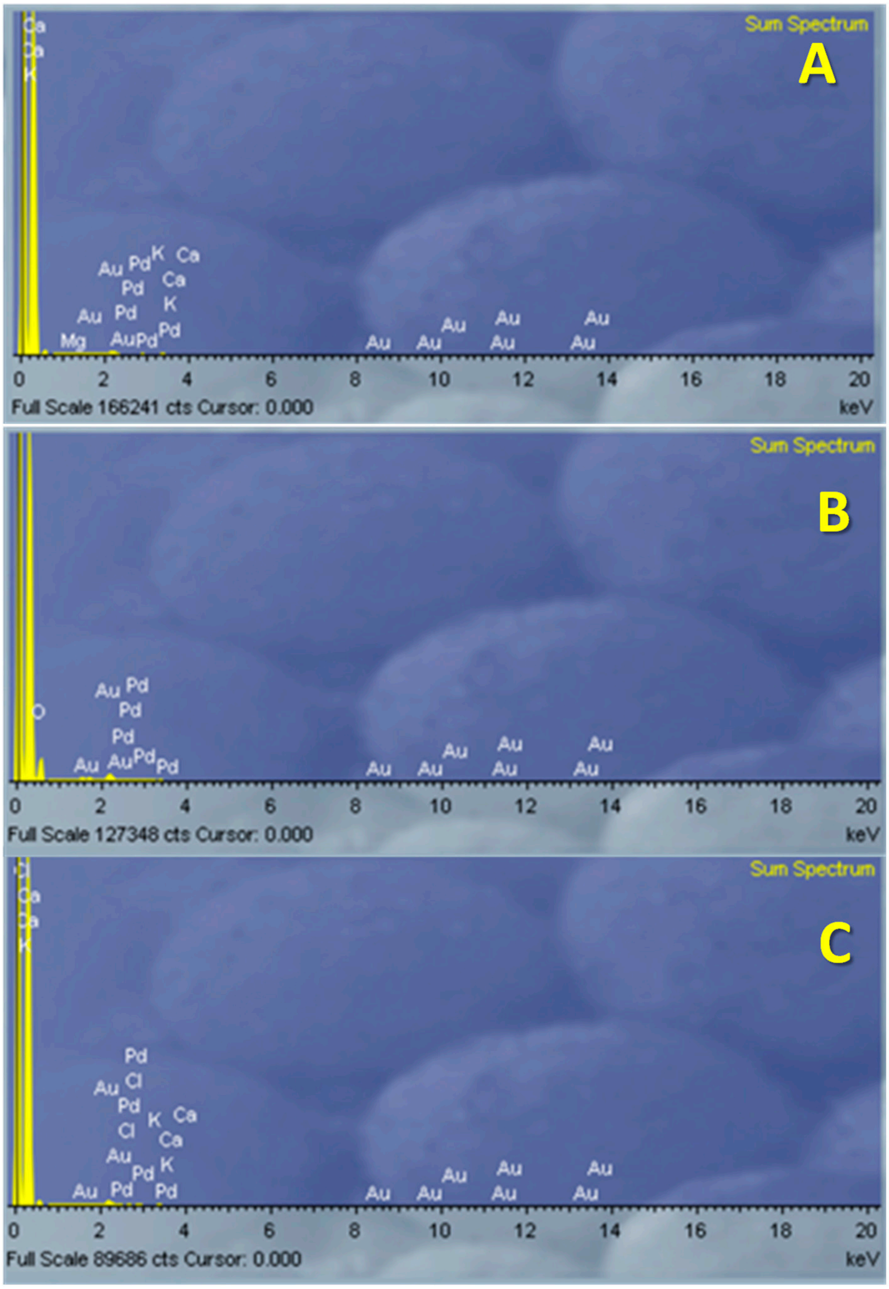

Figure S2. EDX mapping of pristine (A) and modified biochar samples (B, Hummers, and C, persulfate intercalation)

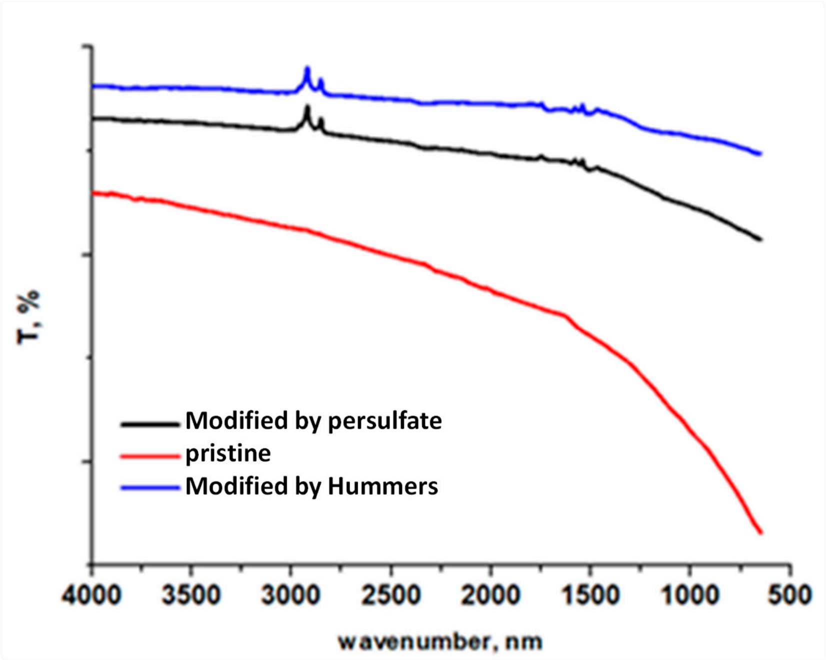

Figure S3. FTIR spectra of pristine and biochar samples modified by Hummers and persulfate intercalation
